# Thermal Lattice Boltzmann Flux Solver for Natural Convection of Nanofluid in a Square Enclosure

**DOI:** 10.3390/e24101448

**Published:** 2022-10-11

**Authors:** Xiaodi Wu, Song Zhou

**Affiliations:** 1 School of Ocean Engineering and Technology, Sun Yat-sen University, Zhuhai 519082, China; 2 School of Aerospace Engineering and Applied Mechanics, Tongji University, Shanghai 200092, China

**Keywords:** natural convection, nanofluid, thermal lattice Boltzmann flux solver, immersed boundary method

## Abstract

In the present study, mathematical modeling was performed to simulate natural convection of a nanofluid in a square enclosure using the thermal lattice Boltzmann flux solver (TLBFS). Firstly, natural convection in a square enclosure, filled with pure fluid (air and water), was investigated to validate the accuracy and performance of the method. Then, influences of the Rayleigh number, of nanoparticle volume fraction on streamlines, isotherms and average Nusselt number were studied. The numerical results illustrated that heat transfer was enhanced with the augmentation of Rayleigh number and nanoparticle volume fraction. There was a linear relationship between the average Nusselt number and solid volume fraction. and there was an exponential relationship between the average Nusselt number and *Ra*. In view of the Cartesian grid used by the immersed boundary method and lattice model, the immersed boundary method was chosen to treat the no-slip boundary condition of the flow field, and the Dirichlet boundary condition of the temperature field, to facilitate natural convection around a bluff body in a square enclosure. The presented numerical algorithm and code implementation were validated by means of numerical examples of natural convection between a concentric circular cylinder and a square enclosure at different aspect ratios. Numerical simulations were conducted for natural convection around a cylinder and square in an enclosure. The results illustrated that nanoparticles enhance heat transfer in higher Rayleigh number, and the heat transfer of the inner cylinder is stronger than that of the square at the same perimeter.

## 1. Introduction

Natural convection has received widespread attention by many researchers because it is relevant to many engineering applications, such as heat exchangers, solar energy and nuclear reactors. Conventional fluids, such as water and ethylene glycol mixture, are not effective heat transfer medias, due to low thermal conductivity. Therefore, nanofluids have gained attention as an alternative and effective heat transfer medium, due to having higher thermal conductivities [1]. There are two main research approaches for studying nanofluids: experiments and numerical simulations. In view of experiments, Song et al. [2] measured the thermal performance of SiC nanofluid in a water pool boiling experiment, and investigated the enhancement for critical heat flux. Nikhah et al. [3] carried out an experimental investigation on the convective boiling of dilute CuO-water nanofluids in an upward flow inside a conventional heat exchanger. Alkasmoul et al. [4] investigated the turbulent flow of Al_2_O_3_-water, TiO_2_-water and CuO-water nanofluids in a heated, horizontal tube with a constant heat flux. The results showed that the efficiency of nanofluids in enhancing heat transfer was not high for turbulent flows. Qi et al. [5] carried out an experimental study on boiling heat transfer of an α-Al_2_O_3_-water nanofluid.

More researchers have applied numerical methods to study the performance of nanofluids. Khanafer et al. [6] directly solved the macroscopic governing equations to investigate heat transfer enhancement in a two-dimensional enclosure utilizing nanofluids for various pertinent parameters, including Grashof numbers and volume fractions. The results indicated that heat transfer increased with the volumetric fraction of the copper nanoparticles in water at any given Grashof number. Fattahi et al. [7] carried out a study on water-based nanofluid, containing Al_2_O_3_ or Cu nanoparticles, in a square cavity for Rayleigh number 10^3^–10^6^ and solid volume fraction 0–0.05, by means of the lattice Boltzmann method. The results indicated that the average Nusselt number increased by increasing the solid volume fraction and the effects of solid volume fraction on Cu were stronger than on Al_2_O_3_. He et al. [8] applied the single-phase lattice model to simulate convection heat transfer utilizing Al_2_O_3_-water nanofluid in a square cavity. Qi et al. [9] applied the two-phase lattice Boltzmann model for natural convection of nanofluid. From the above analysis, the lattice Boltzmann method (LBM) has obtained remarkable achievements in simulating incompressible viscous laminar nanoflow. Saadat et al. [10] developed a compressible LB model on standard lattices to solve supersonic flows involving shock waves, based on the consistent D2Q9 LB model, and with the help of appropriate correction terms introduced into the kinetic equations to compensate for deviations in the hydrodynamic limit. Huang et al. [11] improved the lattice Boltzmann model with a self-tuning equation of state to simulate the thermal flows beyond the Boussinesq and ideal-gas approximations. Hosseini et al. [12] derived the appropriate form of the correction term for the space- and time-discretized LB equations, through a Chapman–Enskog analysis for different orders of the equilibrium distribution function. As a mesoscopic approach, LBM can easily solve the macroscopic variables used by distribution functions and the linear streaming and collision processes can effectively simulate the nonlinear convection and diffusion effects in the macroscopic state. With the development of Lattice models in recent years, LBM can solve various flow problems successfully, including incompressible, compressible and thermal flows, by introducing a variety of applicable models. However, the solutions of flow for High Mach number and turbulence problems of complex shape are limited because the standard LBM is strictly limited to using the uniform Cartesian mesh due to the lattice uniformity for flow.

Recently, the idea of coupling the LBM and conventional methods (including finite difference method and finite volume method) has been proposed for computational fluid dynamics. It effectively combines the merits of macroscopic and mesoscopic methods. The coupling algorithm can be divided into the whole region coupling algorithm and the partition coupling algorithm. The whole region coupling algorithm solves the different variables used by different numerical algorithms. Nie et al. [13] and Mezrhab et al. [14] used the LBM-FDM coupling method to solve natural convection problems, in which LBM solved flow problems and FDM analyzed heat transfer. Chen et al. [15] used the LBM-FDM coupling method to solve the two-phase interface convection problem, in which LBM solved the velocity field and FDM solved the concentration field. Mishra et al. [16] used LBM-FVM to solve heat conduction and radiation problems. Sun and zhang [17] used LBM-FVM for conduction and radiation in irregular geometry. The partition coupling algorithm divides the whole region into several sub-regions and realizes the coupling function through information transfer between the sub-regions. Luan et al. [18,19,20] simulated complex flows in porous media using LBM-FVM. Chen et al. [21,22,23] used LBM-FVM to study the multi-scale flow, multi-component mass transfer, proton conduction and electrochemical reaction processes. Li et al. [24,25] used LBM-FVM to study natural convection and the solid–liquid variation problem. Feng et al. [26] developed a thermal lattice Boltzmann model with a hybrid recursive regularization collision operator on standard lattices for simulation of subsonic and sonic compressible flows without shock by LBM-FVM. Essentially, the main advantage of the above two coupling methods is to improve the calculation efficiency of LBM and expand the applications of macroscopic computational fluid dynamics.

A new coupling idea gas been proposed in the past five years. This coupling method adopts the finite volume method to discretize macroscopic governing equations and uses local lattice Boltzmann equation solutions to calculate interface flux, on the basis of considering migration and collision processes. This method realizes the coupling of the macroscopic method and the mesoscopic model and is named the lattice Boltzmann flux solver (LBFS). Yang et al. [27,28] proposed LBFS based on compressible models, which is suitable for calculating viscous and compressible multi-component flows. Shu et al. [29] and Wang et al. [30,31,32] developed LBFS for incompressible viscous flow problems. This method integrates the advantages of the macroscopic method and the mesoscopic model, to not only realize the unified solution of non-viscous flux and viscous flux, but also to improve calculation efficiency without using a uniform grid in the whole calculation domain. Based on the above development, Wang et al. [33] developed the thermal lattice Boltzmann flux solver (TLBFS) and successfully used it to simulate the natural convection problem. Cao [34] proposed a variable property-based lattice Boltzmann flux solver (VPLBFS) for thermal flows with partial or total variation in fluid properties in the low Mach number limit.

In this paper, we attempted to build mathematical modeling to simulate the natural convection of Al_2_O_3_/water nanofluid in a square enclosure using the thermal lattice Boltzmann flux solver (TLBFS), which is a coupling method combining the finite volume method to discretize the macroscopic governing equations in space, and reconstructed flux solutions at the interface between two adjacent cell centers by using the single-relaxation-time Lattice Boltzmann model. The top mpotivating priority of this paper was to establish a simple and effective numerical calculation method to solve natural convection problems. Therefore, it was necessary to introduce the boundary treatment technique in the solver. Tong et al. [35] applied the multiblock lattice Boltzmann method with a fixed Eulerian mesh, and the fouling layer was represented by an immersed boundary with Lagrangian points. The shape change of the fouling layer could be carried out by deforming the immersed boundary, while keeping the mesh of flow simulation unchanged. Suzuki et al. [36] simulated lift and thrust generation by a butterfly-like flapping wing body model by means of immersed boundary lattice Boltzmann simulations. The immersed boundary method is an effective and simple method to treat solid surface boundary conditions and the numerical method based on a non-body-fitted grid can avoid the abundant work involved in grid generation. Therefore, the immersed boundary method was applied to implement the no-slip boundary condition and Dirichlet boundary condition was applied for natural convection around a bluff body in a square enclosure with the purpose of effective treatment of surface boundaries. Natural convection problems were investigated at different Rayleigh numbers and nanoparticle volume fractions. Influences of the Rayleigh number and nanoparticle volume fraction on the streamlines, isotherms and average Nusselt number were studied.

## 2. Governing Equations and Numerical Method

### 2.1. The Macroscopic Governing Equations

For incompressible thermal nanofluid, in consideration of single phase and constant properties flow conditions, the macroscopic governing equations of natural convection in a two-dimensional enclosure can be written as follows:

Continuity equation;

(1) $$\frac{\partial \rho_{nf}}{\partial t}+\nabla \cdot \rho_{nf}u=0$$

Momentum equation;

(2) $$\frac{\partial }{\partial t}\left(\rho_{nf}u\right)+\nabla \left(\rho_{nf}uu\right)=-\nabla p+\mu_{nf}\nabla \left[\left(\nabla u+{\left(\nabla u\right)}^{T}\right)\right]+F_{nf}$$

Energy equation;

(3) $$\frac{\partial }{\partial t}\left(\rho_{nf}e\right)+\nabla \left(\rho_{nf}ue\right)=\chi_{nf}\nabla^{2}\left(\rho_{nf}e\right)$$

where *ρ*, ***u***, *p* and *m* represent fluid density, velocity, pressure, dynamic viscosity coefficient, respectively; *e* stands for internal energy defined as *e* = *DRT*/2, where *D* is the dimension, *R* is the gas constant and *T* represents the temperature; *χ* is the thermal diffusivity. The subscript *nf* denotes the nanofluid.

Natural convection heat transfer in nanofluids is studied in a two-dimensional enclosure. Nanoparticles considered to be spherical and frictional forces are neglected. The flow is assumed as laminar with a single-phase homogeneous mixture. The buoyancy force always plays an essential role as an external force. Using the Boussinesq approximation, the force source term can be defined as:

(4) $$F_{nf}=\rho_{nf}\beta_{nf}g\left(T-T_{m}\right)j$$

where *g* represents the gravity acceleration, *β* is the thermal expansion coefficient and *T_m_* is the average temperature.

According to Chapman-Enskog analysis, the relationships can be established between the fluxes and the distribution functions of the lattice Boltzmann model. Based on the thermal lattice Boltzmann flux solver (TLBFS), the governing Equations (1)–(3) can be rewritten as:

(5) $$\frac{\partial \rho_{nf}}{\partial t}+\nabla \cdot \left(\sum_{\alpha }e_{\alpha }f_{\alpha }^{eq}\right)=0$$

(6) $$\frac{\partial \rho_{nf}u}{\partial t}+\nabla \cdot \prod_{1}=F_{nf}$$

(7) $$\frac{\partial \rho_{nf}e}{\partial t}+\nabla \cdot \prod_{2}=0$$

where

(8) $$\prod_{1}=\sum_{\alpha =0}^{N}{\left(e_{\alpha }\right)}_{\beta }{\left(e_{\alpha }\right)}_{\gamma }\left[f_{\alpha }^{eq}+(I-\frac{1}{2\tau_{v}})f_{\alpha }^{neq}\right]$$

(9) $$\prod_{2}=\sum_{\alpha =0}^{N}e_{\alpha }\left[g_{\alpha }^{eq}+(I-\frac{1}{2\tau_{c}})g_{\alpha }^{neq}\right]$$

(10) $$\tau_{v}=\mu_{nf}/(\rho_{nf}c_{s}^{2}\delta_{t})+0.5$$

(11) $$\tau_{c}=\chi_{nf}/\left(2c_{s}{}^{2}\delta_{t}\right)+0.5$$

From the above process, the macroscopic flow variables and fluxes can be computed by equilibrium and non-equilibrium distribution functions of the lattice model for the governing equations of nanofluid. Equations (8) and (9) are used to solve the macroscopic flow variables, and fluxes can be evaluated by the thermal lattice Boltzmann flux solver, which is introduced in detail in the next section. The force source term is added at the cell center during the calculation process.

### 2.2. Thermal Lattice Boltzmann Flux Solver

The discrete term of the governing Equations (5)–(7) by finite volume method:

(12) $$\frac{dW_{i}}{dt}=\frac{1}{\Delta V_{i}}\sum_{k}R_{k}dS_{k}+F$$

where $W={\left[\rho_{nf},\rho_{nf}u,\rho_{nf}v,\rho_{nf}e\right]}^{T}$; *dV_i_* and *dS_k_* are the volume of *i*th control volume and the area of the *k*th interface. For the 2D case, the D2Q9 lattice velocity model [37] is used for momentum and energy fluxes. The expression of the fluxes ***R**_k_* at the cell interfaces is as followed:

(13) Rk=(nxf1eq−f3eq+f5eq−f6eq−f7eq+f8eq+nyf2eq−f4eq+f5eq+f6eq−f7eq−f8eqnxf1^+f3^+f5^+f6^+f7^+f8^+nyf5^−f6^+f7^−f8^nxf5^−f6^+f7^−f8^+nyf2^+f4^+f5^+f6^+f7^+f8^nxg1^−g3^+g5^−g6^−g7^+g8^+nyg2^−g4^+g5^+g6^−g7^−g8^)

(14) fα^=fαeq+(1−12τv)fαneq

(15) gα^=gαeq+(1−12τc)gαneq

From Equations (13)–(15), it can be seen that the important segment to solve fluxes is to accurately evaluate the $f_{\alpha }^{eq}$, fα^ and gα^ terms.

The simplified thermal lattice Boltzmann model with BGK approximation can be written as:

(16) $$f_{\alpha }\left(r+e_{\alpha }\delta_{t},t+\delta_{t}\right)-f_{\alpha }\left(r,t\right)=-\frac{1}{\tau_{v}}\left[f_{\alpha }\left(r,t\right)-f_{\alpha }^{eq}\left(r,t\right)\right]$$

(17) $$g_{\alpha }\left(r+e_{\alpha }\delta_{t},t+\delta_{t}\right)-g_{\alpha }\left(r,t\right)=-\frac{1}{\tau_{c}}\left[g_{\alpha }\left(r,t\right)-g_{\alpha }^{eq}\left(r,t\right)\right]$$

In which equilibrium density distribution function and equilibrium internal energy distribution function is given as:

(18) $$f_{\alpha }^{eq}\left(r,t\right)=\rho w_{\alpha }\left[1+\frac{e_{\alpha }\cdot u}{c_{s}^{2}}+\frac{{\left(e_{\alpha }\cdot u\right)}^{2}-{\left(c_{s}\left|u\right|\right)}^{2}}{2c_{s}^{4}}\right]$$

(19) $$g_{\alpha }^{eq}\left(r,t\right)=\left\{\begin{array}{cc} -\frac{2\rho }{3}\frac{{\left|u\right|}^{2}}{c^{2}}, & \alpha =0\\ \frac{\rho e}{9}\left[\frac{3}{2}+\frac{3}{2}\cdot \frac{e_{\alpha }\cdot u}{c^{2}}+\frac{9}{2}\cdot \frac{{\left(e_{\alpha }\cdot u\right)}^{2}}{c^{4}}-\frac{3}{2}\cdot \frac{{\left|u\right|}^{2}}{c^{2}}\right], & \alpha =1,2,3,4\\ \frac{\rho e}{36}\left[3+6\cdot \frac{e_{\alpha }\cdot u}{c^{2}}+\frac{9}{2}\cdot \frac{{\left(e_{\alpha }\cdot u\right)}^{2}}{c^{4}}-\frac{3}{2}\cdot \frac{{\left|u\right|}^{2}}{c^{2}}\right], & \alpha =5,6,7,8 \end{array}\right.$$

Using the second-order Taylor series expansion, Equations (16) and (17) can be transformed as below:

(20) $$\delta_{t}\left(\frac{\partial }{\partial t}+e_{\alpha }\cdot \nabla \right)f_{\alpha }+\frac{\delta_{t}^{2}}{2}{\left(\frac{\partial }{\partial t}+e_{\alpha }\cdot \nabla \right)}^{2}f_{\alpha }+\frac{1}{\tau }\left(f_{\alpha }-f_{\alpha }^{eq}\right)+O\left(\delta_{t}^{3}\right)=0$$

(21) $$\delta_{t}\left(\frac{\partial }{\partial t}+e_{\alpha }\cdot \nabla \right)g_{\alpha }+\frac{\delta_{t}^{2}}{2}{\left(\frac{\partial }{\partial t}+e_{\alpha }\cdot \nabla \right)}^{2}g_{\alpha }+\frac{1}{\tau }\left(g_{\alpha }-g_{\alpha }^{eq}\right)+O\left(\delta_{t}^{3}\right)=0$$

By the multi-scale Chapman-Enskog expansion, the distribution function, the temporal and spatial derivatives, the non-equilibrium density and energy distribution functions can be transformed into an expression only related to the equilibrium distribution functions and can be derived from:

(22) $$f_{\alpha }^{neq}\left(r,t\right)=-\tau_{v}\left[f_{\alpha }^{eq}\left(r,t\right)-f_{\alpha }^{eq}\left(r-e_{\alpha }\delta_{t},t-\delta_{t}\right)\right]$$

(23) $$g_{\alpha }^{neq}\left(r,t\right)=-\tau_{c}\left[g_{\alpha }^{eq}\left(r,t\right)-g_{\alpha }^{eq}\left(r-e_{\alpha }\delta_{t},t-\delta_{t}\right)\right]$$

**Figure 1:**
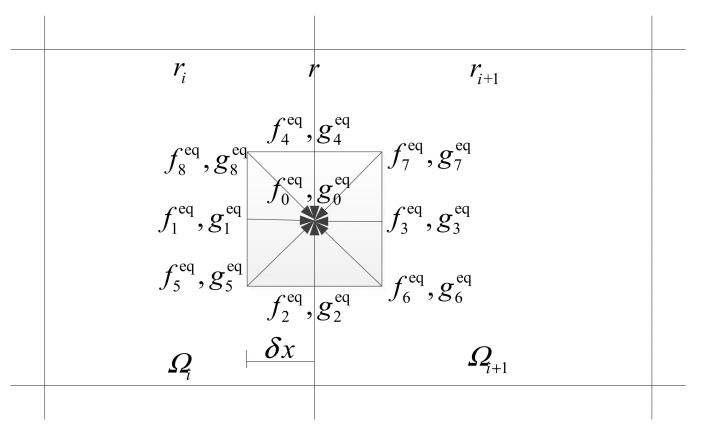
Local flux reconstruction at cell interface.

From Figure 1, the flow properties of eight vertices of the D2Q9 model can be evaluated by interpolation with the given flow properties at the cell centers of two adjacent control volumes. The values ***r****_i_*, ***r**_i_*_+1_ and ***r*** are defined as the physical positions of the two cell centers and their interfaces, respectively. The interpolation formulation can be given as:

(24) $$\psi \left(r-e_{\alpha }\delta_{t},t-\delta_{t}\right)=\left\{\begin{array}{c} \psi \left(r_{i}\right)+\left(r-e_{\alpha }\delta_{t}-r_{i}\right)\cdot \nabla \psi \left(r_{i}\right)\;\;\;\;\;\;\;r-e_{\alpha }\delta_{t}\;in\;\Omega_{i}\\ \psi \left(r_{i+1}\right)+\left(r-e_{\alpha }\delta_{t}-r_{i+1}\right)\cdot \nabla \psi \left(r_{i+1}\right)\;\;\;\;r-e_{\alpha }\delta_{t}\;in\;\Omega_{i+1} \end{array}\right.$$

where ψ stands for the flow properties, including *ρ*, *u*, *v* and *e*. $f_{\alpha }^{eq}\left(r-e_{\alpha }\delta_{t},t-\delta_{t}\right)$ and $g_{\alpha }^{eq}\left(r-e_{\alpha }\delta_{t},t-\delta_{t}\right)$ can be obtained by the corresponding equilibrium density distribution function and energy distribution function. Then, the flow properties of the cell interface can be written as:

(25) $$\rho \left(r,t\right)=\sum_{\alpha =0}f_{\alpha }^{eq}\left(r-e_{\alpha }\delta_{t},t-\delta_{t}\right)$$

(26) $$\rho \left(r,t\right)u\left(r,t\right)=\sum_{\alpha =0}e_{\alpha }f_{\alpha }^{eq}\left(r-e_{\alpha }\delta_{t},t-\delta_{t}\right)$$

(27) $$\rho \left(r,t\right)e\left(r,t\right)=\sum_{\alpha =0}g_{\alpha }^{eq}\left(r-e_{\alpha }\delta_{t},t-\delta_{t}\right)$$

Next, $f_{\alpha }^{eq}\left(r,t\right)$ and $g_{\alpha }^{eq}\left(r,t\right)$ can also be easily solved by distribution functions. After obtaining the equilibrium distribution functions, the fluxes can be evaluated according to Equation (13).

### 2.3. Computational Sequence

The complete numerical simulation procedures for each time step of the proposed method are summarized below.

1. According to the fluid properties of the nanofluid, determine initial velocity and temperature field;
2. Based on the grid size, identify a streaming time step at each interface and then the single relaxation parameters, including dynamic viscosity and the thermal diffusivity;
3. Apply the D2Q9 model to compute the density and energy equilibrium distribution functions $f_{\alpha }^{eq}\left(r-e_{\alpha }\delta_{t},t-\delta_{t}\right)$ and $g_{\alpha }^{eq}\left(r-e_{\alpha }\delta_{t},t-\delta_{t}\right)$ around the middle point ***r*** of each interface;
4. Compute the macroscopic flow properties of nanofluid at the cell interface and then compute $f_{\alpha }^{eq}\left(r,t\right)$ and $g_{\alpha }^{eq}\left(r,t\right)$ by the equilibrium distribution functions of the D2Q9 model;
5. Compute fα^ and gα^ terms, then the fluxes at the cell interface can be solved by Equation (13);
6. Calculate the force source term and add this term to the fluxes;
7. Solve Equations (5)–(7) to obtain the macroscopic flow properties of the nanofluid;
8. Repeat steps (3)–(7) until the following convergence criterion is satisfied.

## 3. Numerical Examples of Natural Convection in a Square Enclosure

### 3.1. Problem Description

**Figure 2:**
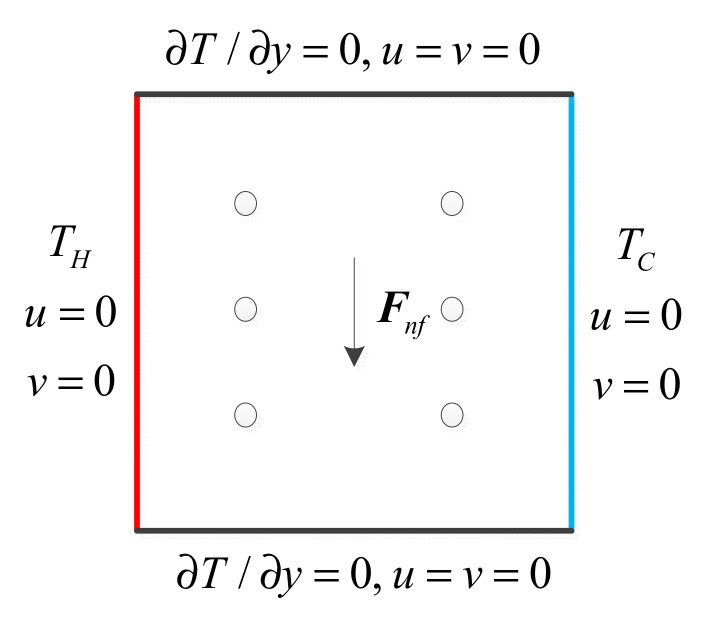
Computational domain and boundary conditions.

The computational domain and boundary conditions are shown in Figure 2. From this figure, it can be seen that the no slip boundary condition was applied on four walls. The adiabatic condition was set on the top and bottom walls and temperatures of 1 and 0 were applied on the left and right walls, respectively. The non-dimensional parameters, Prandtl number *Pr* and Rayleigh number *Ra*, were applied to determine the dynamic similarity as follows:

(28) Pr=ν/χ

(29) $$Ra=\frac{V_{c}^{2}\cdot L^{2}}{\nu \cdot \chi }$$

where *L* = 1 is the characteristic length of the square cavity and *V_c_* is the characteristic thermal velocity which is constrained by the low Mach number limit. In the present simulations, *V_c_* = 0.1 was set in order to ensure incompressible viscous flow.

In the present study, Al_2_O_3_/water nanofluid was used. The thermophysical properties of the water and nanoparticles are listed in Table 1. The homogeneous model for nanofluid was adopted. Physical properties of the nanofluids, including density, specific heat and thermal expansion coefficient, were obtained using the classical formula developed for conventional solid–liquid mixtures as follows:

(30) $$\rho_{nf}=\left(1-\phi \right)\rho_{f}+\phi \rho_{s}$$

(31) $${\left(\rho c_{p}\right)}_{nf}=\left(1-\phi \right){\left(\rho c_{p}\right)}_{f}+\phi {\left(\rho c_{p}\right)}_{s}$$

(32) $$\beta_{nf}=\left(1-\phi \right)\beta_{f}+\phi \beta_{s}$$

where *ϕ* refers to the volume concentration of nanoparticles and the subscripts *s*, *f* denote the particle and base fluids. 

The effective viscosity and thermal conductivity of the nanofluid strongly affect the heat transfer rate and flow characteristics of nanofluids. The effective viscosity could be estimated by experimental correlation for 47 nm Al_2_O_3_/water nanofluid by Angue Mintsa et al. [38] and thermal conductivity was given by Gherasim et al. [39] as follows:

(33) $$\mu_{nf}=0.904e^{14.8\phi }\mu$$

(34) $$\kappa_{nf}=\left(1.72\phi +1.0\right)\kappa_{f}$$

In the present simulations, the convergence criterion for flow field and temperature field were respectively given as follows:

(35) $$Error1=\frac{\sum_{ij}\left|\sqrt{{\left(u\left(i,j,t+\delta_{t}\right)\right)}^{2}+{\left(v\left(i,j,t+\delta_{t}\right)\right)}^{2}}-\sqrt{{\left(u\left(i,j,t\right)\right)}^{2}+{\left(v\left(i,j,t\right)\right)}^{2}}\right|}{\sum_{ij}\sqrt{{\left(u\left(i,j,t+\delta_{t}\right)\right)}^{2}+{\left(v\left(i,j,t+\delta_{t}\right)\right)}^{2}}}\leq 1\times 10^{-7}$$

(36) $$Error2=\frac{\sum_{ij}\left|T\left(i,j,t+\delta_{t}\right)-T\left(i,j,t\right)\right|}{\sum_{ij}T\left(i,j,t+\delta_{t}\right)}\leq 1\times 10^{-7}$$

### 3.2. Natural Convection of Pure Fluid in a Square Enclosure

To testify as to the accuracy and performance of the lattice Boltzmann flux solver based on the population model, the classical natural convection in a square enclosure filled with air and water was studied at *Ra* = 10^3^, 10^4^, 10^5^ and 10^6^.

Firstly, a grid independent study was conducted on five different uniform grids of 101 × 101, 151 × 151, 201 × 201, 251 × 251 and 301 × 301 for the natural convection problem at *Ra* = 10^6^ and *Pr* = 0.7. As shown in Table 2, when the mesh size was 201 × 201, or even larger, the average Nusselt number did not change much and the value was between the benchmark solutions of Davis [40] and Hortmann et al. [41]. When the mesh size was larger than 151 × 151, the maximum horizontal velocity on the vertical mid-plane, the maximum vertical velocity on the horizontal mid-plane and their locations were in agreement with the benchmark solutions of Davis [40]. The above results illustrated grid independence on uniform grids of 201 × 201, for the case of *Ra* = 10^6^.

Based on the above results, the grid independent study was conducted on non-uniform grids by using the size of less than 201 × 201. Table 3 shows the numerical results of six different non-uniform grids of natural convection at *Ra* = 10^6^. From this table, the results were close to the data of uniform grids of 201 × 201 when the non-uniform mesh was more than 121 × 121. In order to ensure the accuracy and efficiency of numerical simulations, the non-uniform grid of 141 × 141 was chosen to simulate natural convection in a square enclosure.

The average Nusselt number results at different Rayleigh numbers are listed in Table 4, and it can be seen that the numerical simulation results were in good agreement with previous literature results at different Rayleigh numbers. This illustrated the accuracy of the present method for natural convection.

**Figure 3:**
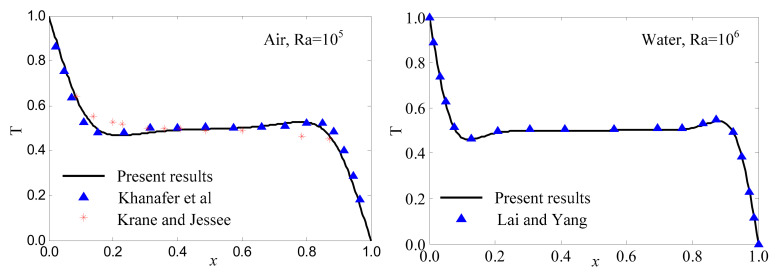
Comparison of temperature distribution at horizontal midsections with previous literatures.

Figure 3 shows the temperature distribution at horizontal midsections of the enclosure. For the enclosure filled with air, the results of *Ra* = 10^5^ were compared with the numerical results of Khanafer et al. [6] and the experimental results of Krane and Jessee [43]. For the enclosure filled with water, the results were compared with numerical results of Lai and Yang [1]. It was noted from the comparisons that the solutions were in excellent agreement. This illustrated that the method in this paper could capture the temperature field very well.

**Figure 4:**
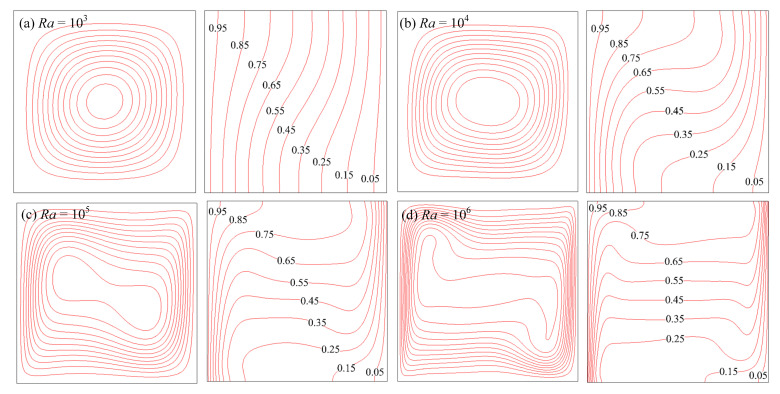
Streamlines and isotherms of air at various Rayleigh numbers.

**Figure 5:**
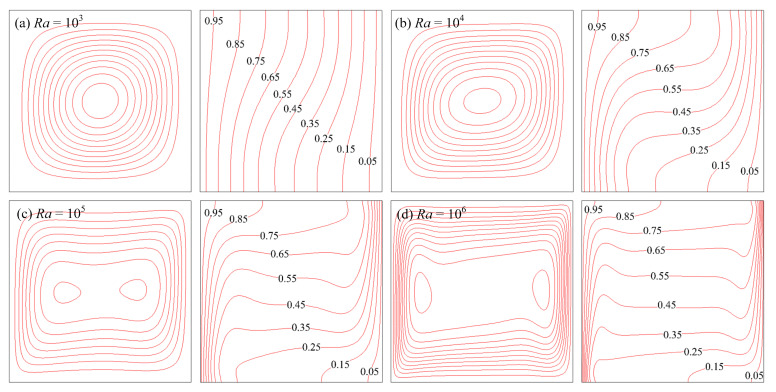
Streamlines and isotherms of water at various Rayleigh numbers.

The streamlines and isotherms of air and water at various Rayleigh numbers are shown in Figure 4 and Figure 5, respectively. It can be seen that the natural convection and heat transfer between the wall and fluid were enhanced as *Ra* increased. For *Ra* ≤ 10^4^, the flow characteristic was to appear as a central vortex. For *Ra* > 10^4^, the central vortex became more expanded and finally broke up into two vortices so that temperature boundary layers were formed. The above phenomenon agreed well with previous studies.

### 3.3. Natural Convection of Nanofluid in a Square Enclosure

After validating the numerical method for natural convection in a square enclosure filled with pure fluid, the natural convection in a square enclosure filled with Al_2_O_3_-water nanofluid of nanoparticles having volume fraction *ϕ* = 1–4% at *Ra* = 10^3^–10^6^ was simulated to validate the present numerical algorithm. The presented averaged Nusselt numbers were compared with the numerical results of Lai and Yang [1] and listed in Table 5. It shows that there was a good agreement and the relative errors were less than 0.8%, which further illustrated that the present numerical method could simulate the natural convection of nanofluid at different Rayleigh numbers and nanoparticle volume fractions.

**Figure 6:**
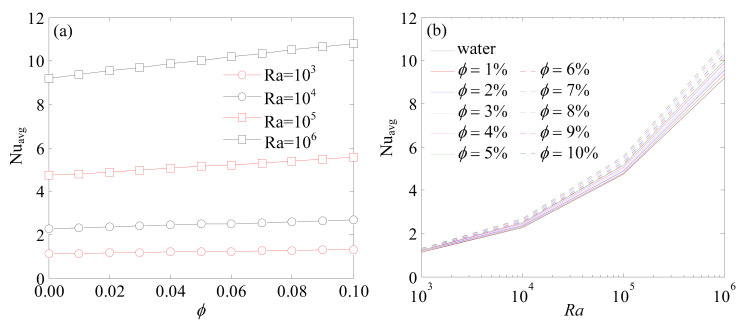
Variation of average Nusselt number against (**a**) solid volume fraction for different Rayleigh number; (**b**) Rayleigh number for different solid volume fraction.

In the present numerical simulations, the effect of nanoparticle suspensions (Al_2_O_3_-water) on flow and temperature characteristics for *Ra* = 10^3^–10^6^ and nanoparticles volume fraction *ϕ* = 0–10% were studied. The variation of average Nusselt number against solid volume fraction for different Rayleigh numbers is shown in Figure 6a and the variation of average Nusselt number against Rayleigh number for different solid volume fractions is shown in Figure 6b.

Numerical results indicated that average Nusselt number increased with the increase of *Ra* and *ϕ*. This illustrated that the function of heat transfer was enhanced with the augmentation of nanofluid thermal conductivity, which indicated that the major mechanism of heat transfer in flowing fluid was thermal dispersion. At the same *Ra*, the relationship of the average Nusselt number and solid volume fraction was almost linear. At the same solid volume fraction, the relationship of the average Nusselt number and *Ra* presented an exponential form. At higher Rayleigh number, the greater the heat transfer rate that could be obtained.

**Figure 7:**
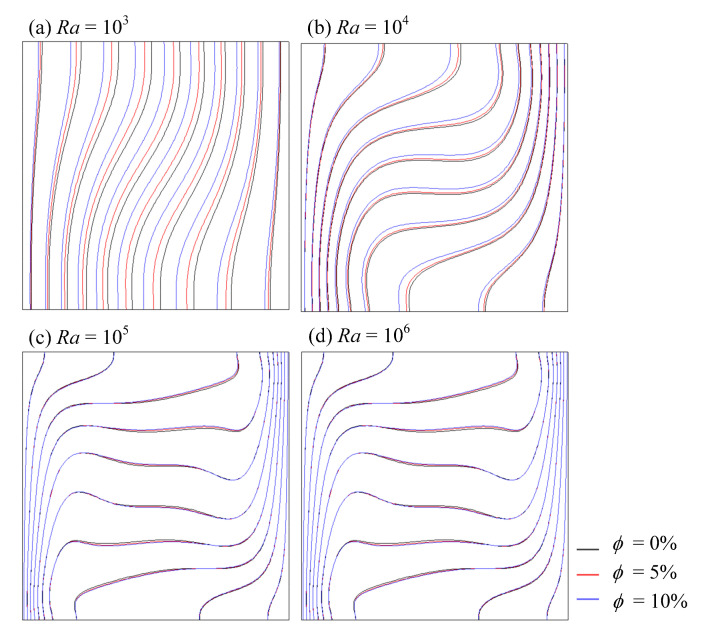
Isotherms of nanofluid at various *Ra* and *ϕ*.

**Figure 8:**
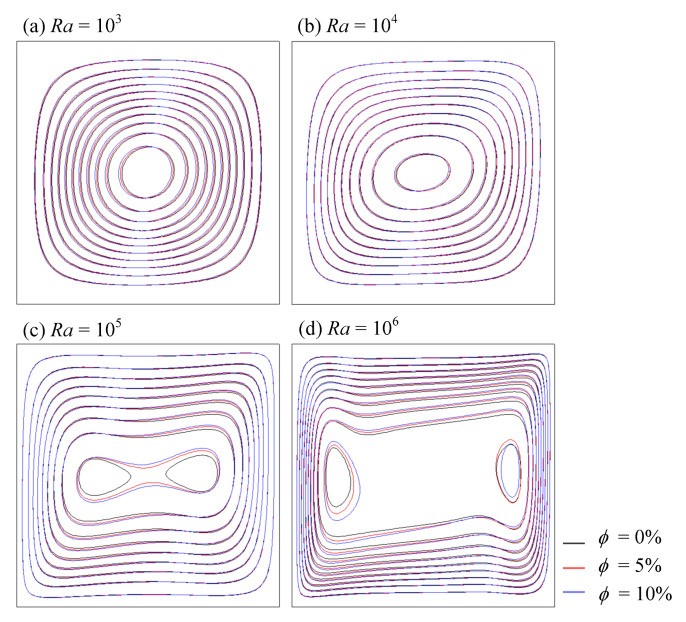
Streamlines of nanofluid at various *Ra* and *ϕ*.

Figure 7 and Figure 8 indicate the isotherms and streamlines of nanofluid (Al_2_O_3_-water) at *Ra* = 10^3^–10^6^ and *ϕ* = 0%, 5% and 10%, which show the effect of volume fraction and *Ra* on flow field and temperature field very well. From Figure 7, it can be seen that heat transfer between the wall and fluid were enhanced as *Ra* increased. As the volume fraction of nanoparticles increased, the isotherm changed slightly. That was because the mixture flow became more viscous, due to the nanoparticles. The velocity of flow fluid reduced and then natural convection weakened. However, the function of heat transfer in total computational domain was enhanced, which was attributed to the augmentation of nanofluid thermal conductivity.

From Figure 8, it can be observed that the flow appeared as a central vortex for lower *Ra*. As *Ra* increased, the central vortex became more expanded and finally broke up into two vortices, so that temperature boundary layers were formed. For pure fluid, the vortex formed in the enclosure as a result of the buoyancy effect. By increasing the volume fraction of nanoparticles, the intensity of streamlines increased, due to the high energy transport through the flow as a result of irregular motion of the ultra-fine particles.

## 4. Numerical Examples of Natural Convection around Bluff Body in a Square Enclosure

### 4.1. Problem Description

The boundary condition-enforced immersed boundary method was chosen for treatment of the solid boundary conditions. Based on the immersed boundary method and thermal lattice Boltzmann flux solver (IB-TLBFS), the macroscopic governing equations can be rewritten as:

(37) $$\frac{\partial \rho_{nf}}{\partial t}+\nabla \cdot \left(\sum_{\alpha }e_{\alpha }f_{\alpha }^{eq}\right)=0$$

(38) $$\frac{\partial \rho_{nf}u}{\partial t}+\nabla \cdot \prod_{1}=F_{nf}+f_{b}$$

(39) $$\frac{\partial \rho_{nf}e}{\partial t}+\nabla \cdot \prod_{2}=q_{b}$$

where the force source term $f_{b}$ and the heat source term *$q_{b}$* are both generated by the immersed boundary. To solve the governing equations, the calculation process is divided into two steps: the first step predicts the state variables without taking account of the boundary function and the second step corrects velocity and temperature by the immersed boundary method.

In this work, the implicit velocity correction scheme proposed by Wang et al. [44] was be applied in view of satisfaction of the no slip boundary. The implicit heat source scheme proposed by Ren et al. [45] was applied for the Dirichlet boundary conditions of the temperature field.

**Figure 9:**
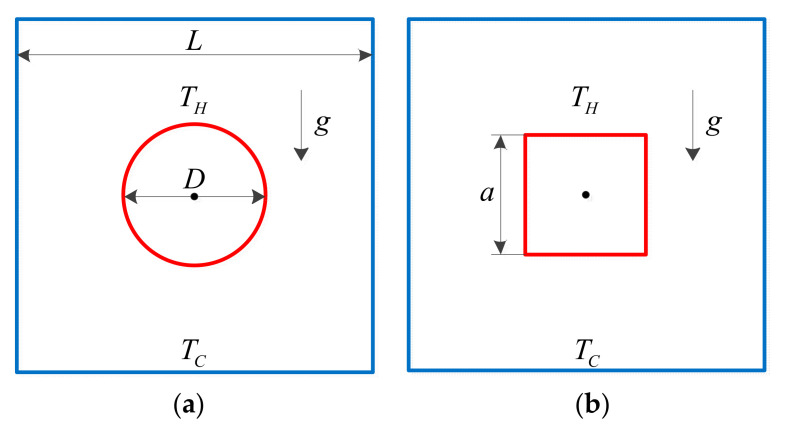
The physical models, computational domain and boundary conditions between bluff body and square enclosure. (**a**) Cylinder (**b**) Square.

Natural convection of a heated bluff body in a square enclosure was studied. The physical models, computational domain and boundary conditions are presented in Figure 9. All boundaries were no-slip and isothermal boundary conditions. The flow was assumed to be laminar and driven by the temperature difference.

Numerical investigations were carried on two types of bluff bodies, a circular cylinder and a square. The four side walls of the outer square enclosure were cooled isothermally at *T_C_* and the side length was *L*. The wall of the inner bluff body was heated isothermally at *T_H_* and *D* and *a* represent the diameter of the circular cylinder and the side length of the square, respectively. For fixed Rayleigh number, numerical simulation cases were designed to have a fixed perimeter for different bluff bodies and the influences of geometry on the heat transfer is discussed in detail.

### 4.2. Natural Convection in the Annulus between Concentric Circular Cylinder and Square Enclosure

After validating the numerical algorithm of the thermal lattice Boltzmann flux solver, natural convection in the annulus between concentric circular cylinder and square enclosure at *Ra* = 10^4^, 10^5^ and 10^6^ were simulated to validate the immersed boundary method and code implementation. Numerical simulations were conducted for three different aspect ratios (*Ar* = 1.67, 2.5 and 5.0). The average Nusselt number was also computed and compared with reference data in the literature.

The computed average Nusselt numbers are compared in Table 6 with those of Ren et al. [45], Shu et al. [46] and Moukalled et al. [47]. From this table, it can be seen that the present results of the method combining IBM and TLBFS agreed very well with reference data. Besides this, the results revealed that the average Nusselt number greatly depended on Rayleigh number and aspect ratio. Due to buoyancy-induced convection, the average Nusselt number increased with increase of *Ra*, while it decreased with increase of *Ar*, due to the effect of annulus gap space.

**Figure 10:**
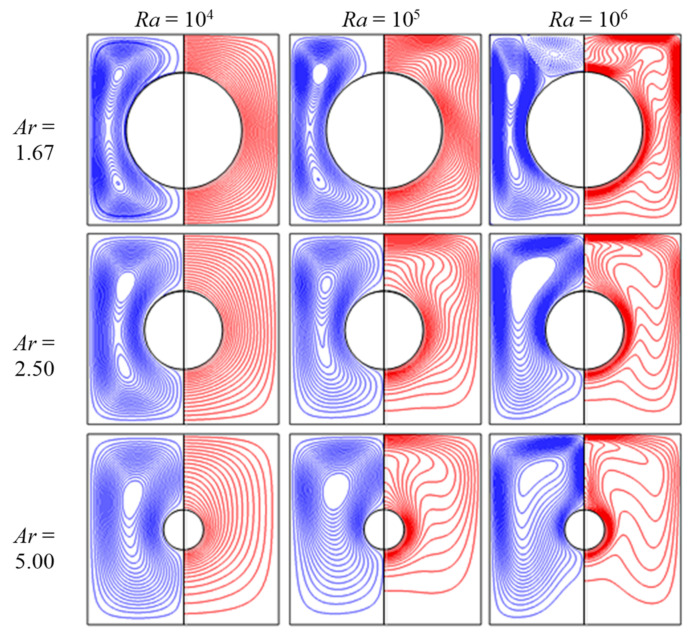
Streamlines and isotherms at various *Ra* and *Ar*.

The streamlines and isotherms in the annulus at various Rayleigh numbers and aspect ratios are shown in Figure 10. Conduction dominated the flow field and a relatively weak convective flow could be observed in the annulus at the lower *Ra*. As the Rayleigh number increased, the strength of the convective flow grew and the center of the recirculation eddy changed its position. When *Ra* = 10^6^, a relatively stronger convective flow dominated the fluid field and a higher temperature gradient could be observed. In contrast, stronger convective flow and higher temperature gradient could be observed in the case of lower values of *Ar*.

### 4.3. Natural Convection of Nanofluid between Bluff Body and Square Enclosure

In the present study, numerical investigations of natural convection between heated bluff body and square enclosure were conducted for nanoparticles having volume fractions of *ϕ* = 0%, 2% and 4% and Rayleigh numbers of *Ra* = 10^4^, 10^5^ and 10^6^.

The averaged Nusselt numbers are listed in Table 7. The numerical simulation results indicated that average Nusselt number increased with increase of *Ra* and *ϕ*, which was the same as occurred in natural convection of square enclosure. By comparison, the averaged Nusselt number of the natural convection around a circular cylinder in an enclosure was greater than that of the square at the same calculation conditions. This illustrated that a smooth geometrical shape was beneficial to heat transfer.

**Figure 11:**
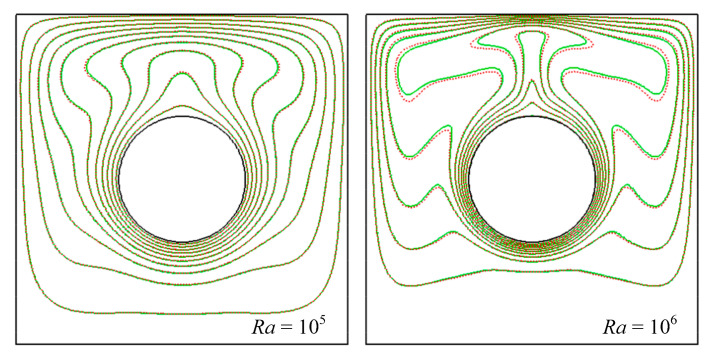
Isotherms for natural convection around cylinder at different nanoparticles volume fraction. Green line: *ϕ* = 0.00, red line: *ϕ* = 0.04.

**Figure 12:**
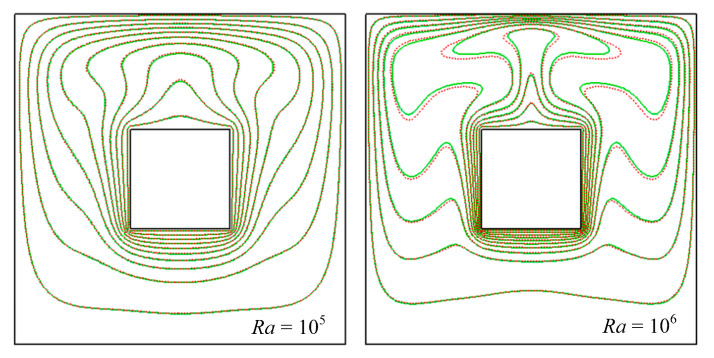
Isotherms for natural convection around square at different nanoparticles volume fraction. Green line: *ϕ* = 0.00, red line: *ϕ* = 0.04.

Figure 11 and Figure 12 present the distribution of the isotherms for different Rayleigh numbers (*Ra* = 10^5^ and 10^6^) and values of nanoparticle volume fractions (*ϕ* = 0 and 0.04). An overview of this figure indicated that the thermal fields strongly depended on Rayleigh number. When *Ra* = 10^5^ or even lower, the isotherms of *ϕ* = 0 were almost close to that of *ϕ* = 0.04, which illustrated that nanoparticle volume fraction played a smaller role in heat transfer and flow pattern. When *Ra* = 10^6^, there were significant differences between the isotherms of *ϕ* = 0 and *ϕ* = 0.04, which illustrated that nanoparticle volume fraction played a role in heat transfer and flow pattern for high *Ra*. The thickness of the thermal boundary layer decreased as the volume fraction increased, which was due to the increasing conduction heat transfer by adding nanoparticle volume fraction.

**Figure 13:**
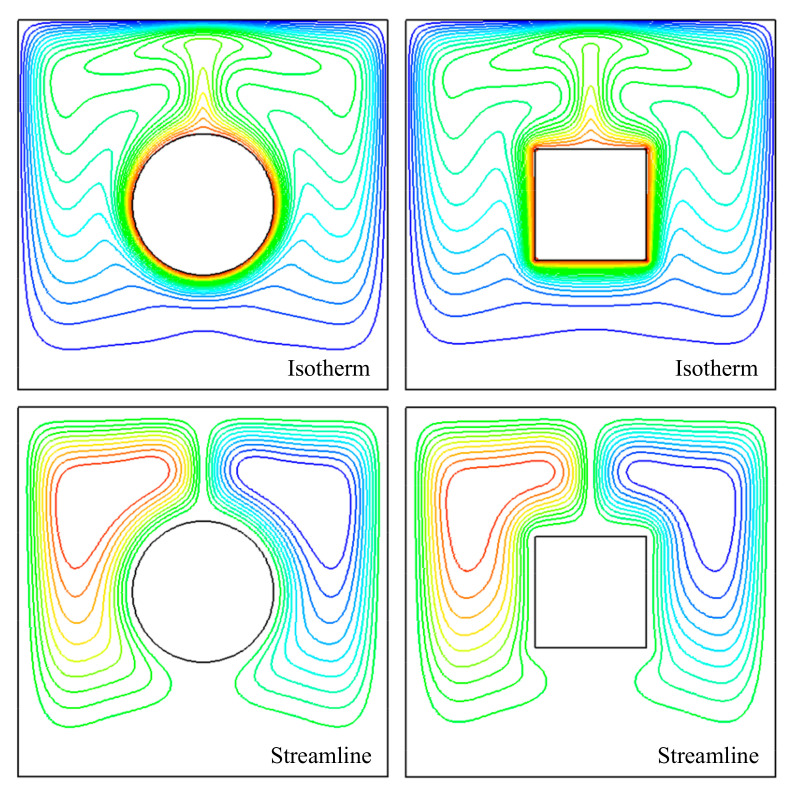
Isotherms and streamlines for natural convection at nanoparticles volume fraction *ϕ* = 0.04 and *Ra* = 10^6^.

Figure 13 shows the streamlines for natural convection around a circular cylinder and square at nanoparticle volume fractions *ϕ* = 0.04 and *Ra* = 10^6^. From Table 7, it can be seen that the preferable heat transfer effect could be acquired by the cylinder in comparison with the square at the same perimeter. That was because the velocity and temperature gradients around the sharp corners of the square dramatically changed, which prevented the heat transfer effect.

## 5. Conclusions

The thermal lattice Boltzmann flux solver (TLBFS) was applied to simulate natural convection of nanofluid in a square enclosure. This method couples the finite volume method and lattice Boltzmann models to realize the solution of incompressible thermal flow. To validate the accuracy and performance of this method, natural convection in a square enclosure filled with pure fluid (air and water) was first studied. There were good agreements with previous literature. Numerical investigations of fluid flow and convective heat transfer were performed. The effects of some parameters, such as the Rayleigh number (*Ra*), and volume fraction of nanoparticles (*ϕ*), on natural convection were analyzed. With increase in the Rayleigh number and nanoparticle volume fraction, the heat transfer rate increased and the nanofluid flow became more viscous and this led to a decrease in nanofluid motion velocity. The average Nusselt number was an increasing exponential function of the Rayleigh number and an increasing linear function of the nanoparticle volume fraction. Then, natural convection around a bluff body in a square enclosure was studied by a method combining TLBFS and immersed boundary method. Natural convection problems in the annulus between concentric circular cylinder and square enclosure without nanofluid were simulated, which validated the feasibility of the numerical algorithm and code implement. Numerical investigations of natural convection between heated bluff body (cylinder and square) and square enclosure were conducted for different nanoparticle volume fractions and Rayleigh numbers. The numerical results illustrated that heat transfer effect increased with increase of *Ra* and *ϕ*. At lower *Ra*, the function of heat transfer with the augmentation of nanofluid thermal conductivity was counteracted by the more viscous flow. Nevertheless, nanoparticles played a better role in enhancing natural convection at higher *Ra*. The above results declare that the TLBFS is a promising method for heat transfer of nanofluids of the future.

## Figures and Tables

**Table 1:** Thermophysical properties of fluid and nanoparticles.

Properties	Fluid Phase (Water)	Solid Phase (Al_2_O_3_)
*ρ* (kg/m^3^)	997	3880
*c_p_* (J/kg·K)	4179	765
*β* (1/K)	0.00021	0.0000085
*k* (W/m·K)	0.613	40
*μ* (kg/m·s)	0.000855	-

**Table 2:** Grid independent study on uniform of natural convection at *Ra* = 10^6^.

Method	*Grids*	*Nu_avg_*	*y*	*u_max_*	*x*	*v_max_*
Present	101 × 101	8.788	0.855	64.22	0.0350	217.60
	151 × 151	8.809	0.850	64.66	0.0367	219.32
	201 × 201	8.816	0.853	64.99	0.0375	220.05
	251 × 251	8.819	0.854	66.18	0.0380	220.17
	301 × 301	8.819	0.852	66.99	0.0383	220.14
De Vahl Davis [40]		8.800	0.850	64.63	0.0397	219.36
Hortmann et al. [41]		8.825	-	-	-	-

**Table 3:** Grid independent study on non-uniform of natural convection at *Ra* = 10^6^.

*Grids*	*Nu_avg_*	*y*	*u_max_*	*x*	*v_max_*
81 × 81	8.803	0.856	64.77	0.0358	218.98
101 × 101	8.811	0.855	64.99	0.0349	219.16
121 × 121	8.816	0.854	65.04	0.0400	219.37
141 × 141	8.818	0.853	65.03	0.0388	220.15
161 × 161	8.819	0.853	65.03	0.0379	220.14
181 × 181	8.820	0.853	65.06	0.0372	220.22

**Table 4:** Comparison of average Nusselt numbers at different Rayleigh numbers.

*Ra*	Air				Water		
	Present	Davis [40]	Khanafer et al. [6]	Qi [9]	Present	Kahveci [42]	Lai and Yang [1]
10^3^	1.118	1.118	1.118	1.118	1.119	-	1.128
10^4^	2.246	2.243	2.245	2.247	2.278	2.274	2.286
10^5^	4.522	4.519	4.522	4.522	4.725	4.722	4.729
10^6^	8.818	8.800	8.826	8.808	9.204	9.230	9.173

**Table 5:** Comparison of average Nusselt numbers with the previous studies.

*Ra*	*ϕ*	Present	Ref [1]	Relative Error (%)
10^3^	0.01	1.139	1.147	0.697
	0.02	1.158	1.167	0.771
	0.03	1.177	1.186	0.756
	0.04	1.196	1.206	0.829
10^4^	0.01	2.317	2.326	0.387
	0.02	2.357	2.366	0.380
	0.03	2.396	2.406	0.416
	0.04	2.435	2.445	0.409
10^5^	0.01	4.807	4.811	0.008
	0.02	4.890	4.894	0.008
	0.03	4.972	4.977	0.010
	0.04	5.054	5.059	0.010
10^6^	0.01	9.366	9.331	0.375
	0.02	9.528	9.492	0.386
	0.03	9.688	9.653	0.363
	0.04	9.849	9.813	0.367

**Table 6:** Comparison of average Nusselt numbers at different *Ra* and *Ar*.

*Ra*	*Ar*	Present	Ren et al. [45]	Shu and Zhu [46]	Moukalled and Acharya [47]
10^4^	1.67	5.425	5.303	5.40	5.826
	2.50	3.256	3.161	3.24	3.331
	5.00	2.090	2.051	2.08	2.071
10^5^	1.67	6.285	6.171	6.21	6.212
	2.50	4.954	4.836	4.86	5.080
	5.00	3.809	3.704	3.79	3.825
10^6^	1.67	11.943	11.857	12.00	11.620
	2.50	9.002	8.546	8.90	9.374
	5.00	6.110	5.944	6.11	6.107

**Table 7:** Comparison of average Nusselt numbers at various *Ra* and *ϕ*.

	Cylinder			Square		
*ϕ*	*Ra* = 10^4^	*Ra* = 10^5^	*Ra* = 10^6^	*Ra* = 10^4^	*Ra* = 10^5^	*Ra* = 10^6^
0.00	3.131	5.080	9.144	2.9432	4.8675	8.6541
0.02	3.132	5.098	9.200	2.9447	4.8884	8.7186
0.04	3.134	5.117	9.263	2.9463	4.9103	8.7883
